# Insight into the Functional Diversification of Lipases in the Endoparasitoid *Pteromalus puparum* (Hymenoptera: Pteromalidae) by Genome-scale Annotation and Expression Analysis

**DOI:** 10.3390/insects11040227

**Published:** 2020-04-05

**Authors:** Jiale Wang, Jiqiang Song, Qi Fang, Hongwei Yao, Fang Wang, Qisheng Song, Gongyin Ye

**Affiliations:** 1 State Key Laboratory of Rice Biology & Ministry of Agriculture and Rural Affairs Key Laboratory of Molecular Biology of Crop Pathogens and Insects, Institute of Insect Sciences, Zhejiang University, Hangzhou 310058, China; jialewang@zju.edu.cn (J.W.); jqsong@zju.edu.cn (J.S.); fangqi@zju.edu.cn (Q.F.); hwyao@zju.edu.cn (H.Y.); wangf121@zju.edu.cn (F.W.); 2 Division of Plant Sciences, University of Missouri, Columbia, MO 65211, USA; SongQ@missouri.edu

**Keywords:** triacylglycerol acylhydrolase, parasitoid wasp, annotation, venom, salivary, lipid

## Abstract

Lipases play essential roles in digestion, transport, and processing of dietary lipids in insects. For parasitoid wasps with a unique life cycle, lipase functions could be multitudinous in particular. *Pteromalus puparum* is a pupal endoparasitoid of butterflies. The female adult deposits eggs into its host, along with multifunctional venom, and the developing larvae consume host as its main nutrition source. Parasitoid lipases are known to participate in the food digestion process, but the mechanism remains unclear. *P. puparum* genome and transcriptome data were interrogated. Multiple alignments and phylogenetic trees were constructed. We annotated a total of 64 predicted lipase genes belonging to five lipase families and suggested that eight venom and four salivary lipases could determine host nutrition environment post-parasitization. Many putative venom lipases were found with incomplete catalytic triads, relatively long β9 loops, and short lids. Data analysis reveals the loss of catalytic activities and weak triacylglycerol (TAG) hydrolytic activities of lipases in venom. Phylogenetic trees indicate various predicted functions of lipases in *P. puparum*. Our information enriches the database of parasitoid lipases and the knowledge of their functional diversification, providing novel insight into how parasitoid wasps manipulate host lipid storage by using venom lipases.

## 1. Introduction

Lipases (EC 3.1.1.3), also defined as triacylglycerol (TAG) acylhydrolases, usually catalyze the hydrolysis of lipids at the lipid–water interface and thus play a crucial role in controlling lipid uptake, transport, and utilization in insects. Most lipases from all organisms can be grouped into six families according to the sequence relationship within the α/β hydrolase fold superfamily of proteins. They are neutral (PF00151), acid (PF04083), lipase2 (PF01674), lipase3 (PF01764), GDSL (named after the conserved amino acid motif of Gly, Asp, Ser, and Leu around the active site Ser; PF00657), and hormone-sensitive lipases (PF06350) [1,2]. With a catalytic triad (usually Ser–Asp/Glu–His), which is capable of generating a charge relay system and a highly nucleophilic serine, all lipases from those six families use a two-step reaction mechanism. Besides these six families, phospholipase A2 (EC 3.1.1.4) is another lipase family belonging to a separate superfamily but using a different catalytic mechanism [3]. Although it contains lots of phospholipases, it is not considered or analyzed in this study. 

Each of these six lipase families has a functional responsibility. The neutral lipases can hydrolyze not only neutral lipids such as TAGs, diacylglycerides (DAGs), and monoacylglycerols (MAGs) but also the sn-1 position in phospholipids and galactolipids [1,4]. The acid or abhydrolipase family was named after the function of the gastric lipase of mammals at acidic pH. It mainly hydrolyzes TAGs and cholesterol esters [5]. Lipases from lipase2 and lipase3 families can hydrolyze TAGs or carboxylesters with differing fatty acid elements [2]. GDSL family members can hydrolyze fatty acids from TAGs, DAGs, MAGs, phospholipids with the sn-2 position, and carboxylester or thioester substrates [6]. The hormone-sensitive lipases can hydrolyze TAGs and cholesterol esters [7] but are restrained by hormonal and neuronal controls via reversible cAMP-dependent phosphorylation of a serine residue [8].

*Pteromalus puparum* (Hymenoptera: Pteromalidae) is a pupal endoparasitoid with a wide host range but preferably lays eggs in the pupae of certain papilionid and pieridid butterfly species. Benefitting from its prominent venom, which is co-injected into hosts during oviposition, *P. puparum* can suppress the population of the cabbage butterfly *Pieris rapae* (Lepidoptera: Pieridae) with a parasitism rate greater than 90% in the cruciferous crops fields in China [9]. It has been used as an endoparasitoid model to study the parasitoid–host interaction in recent years, and the compositions of its venom have also been investigated. Combining the proteomics and transcriptomics data of *P. puparum* venom, 70 putative venom proteins have been previously identified [10,11], including a group of venom lipases. Interestingly, the number of predicted lipases in *P. puparum* venom is higher than that in other parasitoid wasps (Supplementary Materials Table S1) [10,12,13,14,15,16,17,18,19,20,21,22]. 

We had previously assumed that *P. puparum* venom lipases might act on catalyzing the hydrolysis of TAG to free fatty acid and glycerol [23,24,25,26] as usual. However, this assumption was questioned by some results of lipidomics analysis, which used a liquid chromatography and mass spectrometry (LC–MS)-based approach to identify and quantify the lipids in the parasitized hosts. The lipidomic analysis revealed that hemolymph TAG levels in *P. rapae* increased while DAGs levels significantly decreased post-parasitization by *P. puparum* [27]. In the host cotton–melon aphid *Aphis gossypii*, which was parasitized by *Lysiphlebia japonica*, levels of TAG mostly increased as well [28]. The accumulation of TAGs in parasitized hosts is antagonistic to the typical function of lipases, implying that lipases participating in adjusting lipid availability in hosts to benefit wasp offspring likely lose TAG activity or have evolved novel functions. Therefore, in this paper, we interrogated the genome and transcriptome of *P. puparum* to annotate the predicted lipases and analyze their expression profiles. We were especially interested in the sequence characteristics of the putative venom lipases and larval salivary lipases since they are responsible for regulating the lipid metabolism in hosts in particular.

To investigate the enzyme activities and potential functions of *P. puparum* lipases, we first identified their catalytic triads. Subsequently, multiple sequence alignments of β9 loop and lid motifs were used to estimate the TAG hydrolytic activity of neutral lipases. Putative functions of *P. puparum* neutral and acid lipases were predicted by phylogeny analysis. Our information provides an overview of the potential lipase functions in *P. puparum*. It also gives novel insights into studying the diversity and evolution of venom/salivary lipases in parasitoid wasps. 

## 2. Materials and Methods

### 2.1. Annotation of Encoding Lipase Genes

Seven lipase protein sequences from the thoroughly annotated *Drosophila melanogaster* genome (flybase.org) were used as probes in *tBLASTn* analysis to identify lipases in Hymenoptera species. The *Pteromalus puparum* genome was sequenced and assembled in our previous work [29]. Other nine genome assemblies of parasitoid wasps, including *Ceratosolen solmsi*, *Copidosoma floridanum*, *Diachasma alloeum*, *Fopius arisanus*, *Nasonia vitripennis*, *Orussus abietinus*, *Trichogramma pretiosum*, and *Solenopsis invicta* were downloaded from NCBI GenBank. The probe CG6283 was used to identify natural lipases; CG6753, CG11598, and LIP (CG7279) for acid lipases; CG11029 for the family GDSL; CG33174 for the family lipase3; and CG11055 for hormone-sensitive lipases. Since lipase2 sequence was not found in *D. melanogaster* and other insect genomes, a *Caenorhabditis elegans* lipase2 (NP_496693) was added and used as a probe of this family (Supplementary file 1). Using the tool HMMER v3.1b2 and Pfam31.0 database [30], we searched the domains of all candidate sequences and filtered sequences as follows: PF00151 (neutral), PF04083 (acid), PF01674 (lipase2), PF01764 (lipase3), PF00657 (GDSL), and hormone-sensitive lipase (PF06350) [1,2]. All predicted lipase genes were manually validated by *BLASTp* (blast.ncbi.nlm.nih.gov/Blast.cgi?PAGE=Proteins), and their signal peptides were predicted by the online program SignalP (www.cbs.dtu.dk/services/SignalP) [31]. 

### 2.2. Expression Patterns Analysis

Our laboratory previously generated a number of RNA-seq libraries from *P. puparum* (eggs, larvae, female pupae, male pupae, female adults, male adults, female venom glands, female carcass without venom glands, ovaries, larval salivary glands) (access to the released transcriptome data via GECT01000000). Trinity and Cufflinks were used to assemble the transcriptomic raw data and calculate the expression levels of genes [32], which were represented as fragments per kilobase of exon model per million mapped fragments (FPKM). It is proportional to the number of cDNA fragments originating from it. Using a tool of the website BMK-Cloud (www.biocloud.net), two heatmap figures displaying expression profiles of lipases across *P. puparum* different developmental stages and tissues were constructed, respectively. The Venn diagram was drawn using an online program (bioinformatics.psb.ugent.be/webtools/Venn/).

### 2.3. RNA Extraction and cDNA Synthesis

To validate the expression patterns of lipases in *P. puparum*, we collected a total of 15 groups of *P. puparum* samples: six sample groups of different developmental stages or genders (embryos, larvae, female pupae, male pupae, female adults, and male adults), three larval tissues (midguts, salivary glands, and carcass), and six female tissues (heads, thoraxes, midguts, ovaries, venom glands, and carcass), as described previously [33]. They were pooled into a centrifuge tube with TRIzol reagent (Invitrogen, Carlsbad, CA, US) separately for the total RNA extractions. The PrimeScript™ One-Step RT-PCR Kit (Takara, Kusatsu, Japan) was subsequently used for reverse transcriptions according to the manufacturer’s instructions, and the synthesized cDNA was stored at −80 °C for further experiments.

### 2.4. RT-qPCR

SYBR Green Supermix Kit (Takara, Kusatsu, Japan) was used to perform a real-time quantitative PCR (RT-qPCR) on the BIO-RAD CFX96™ Real-Time System. RT-qPCR reaction was set up as follows: 95 °C for 30 s, 40 cycles at 95 °C for 5 s to denature DNA, and 60 °C for 34 s to anneal. The relative expression levels were conducted based on the 2^–ΔΔCt^ method [34]. A stably expressed reference gene 18s rRNA was selected as an internal control. All primers used were designed on Primer3web (primer3.ut.ee) (Supplementary Materials Table S2).

### 2.5. Identification of the Catalytic Triads in P. puparum Lipases

To search the catalytic residues of neutral and acid lipase protein sequences in *P. puparum*, sequences of recognized canine pancreatic (NP_001003319) and gastric (NP_001003209) lipases were downloaded from NCBI and used to perform multiple sequence alignments, respectively [35]. Alignments were constructed by the Clustal Omega program (www.ebi.ac.uk/Tools/msa/clustalo), and then displayed and edited by the software GeneDoc. 

### 2.6. Search for the β9 loop and Lid Motifs in P. puparum Lipases

For the analysis of β9 loop and lid motifs, eight neutral lipase sequences from holometabolous insects, namely *Anopheles gambiae* (AgamN14a and AgamN14b), *Apis mellifera* (AmelN9 and AmelN10), *D. melanogaster* (CG5966 and CG6847), *Bombyx mori* (BmorN12), and *Tribolium castaneum* (TcasN6) were used for a sequence alignment with all predicted neutral lipases in *P. puparum*. Sequence alignments were constructed as described above. The β9 loop and lid motifs of neutral lipases in *P. puparum* were identified according to the alignment results. Taking CG5966 as an example, its β9 loop was contained between residues His254–His273 and the lid domain was defined by a disulfide bridge between Cys287 and Cys310.

### 2.7. Phylogenetic Analysis

A number of neutral and acid lipase protein sequences of holometabolous insects [36] were used to analyze the predicted lipases in *P. puparum*. Muscle v3.8.31 was used for alignments, and trimAl v1.2 was used for the automated trimming of multiple sequence alignments [37,38]. Phylogenetic trees were constructed by RAxML with 1000 bootstrap replications [39]. According to the lowest BIC and AIC score conculcated by ProtTest v3.4 [40,41], their best models of amino acid replacement were selected as LG. The online tool iTOL v3 was used for tree visualization [42].

## 3. Results

### 3.1. Genome-Scale Identification of Encoding Lipases in Parasitoid Wasps

A total of 64 genes were identified as potential lipase encoding genes in *P. puparum* on the genome-scale (Supplementary Materials Table S3). Among them, 32 encoded for neutral lipases, 26 for acid lipases, 3 for lipase3, 2 for GDSL, and 1 for hormone-sensitive lipase. Considering the existence of isoforms of the same gene that could be repeatedly counted, here we lined out all genes encoding lipases tandemly arranged on the same scaffold and aligned with the same lipase gene in *N. vitripennis*, a closely related species of *P. puparum* in the same family of Pteromalidae. PPU13509 and PPU13510 on scaffold 13 were both identical to XP_008213053.2, PPU11430 and PPU11431 on scaffold 26 corresponded to XP_016840181.1, PPU09230 and PPU09231 on scaffold 150 to XP_016838373.1, and PPU04615 and PPU04616 on scaffold 0 to XP_016836798.1.

We also annotated the lipase encoding genes of eight parasitoid wasps and one ant species using their genomes to compare the number of each lipase gene family among insect species (Table 1). The total number of lipase genes varied from 26 in *A. mellifera* to 68 in *P. puparum*. *N. vitripennis*, the sister species of *P. puparum*, had a similar number of lipid genes in each family as *P. puparum* and a relatively large total number of predicted lipases (62). *T. pretiosum* had 59 lipases in total. For the other parasitoid wasps, the total number of lipase genes in *D. alloeum, C. floridanum*, and *F. arisanus* were all less than 50 each (48, 46, and 38, respectively). Interestingly, the total number of lipase genes in insect species seems to be related to the diversity of their food. There were 31 lipase genes found in the fig wasp *C. solmsi,* which lays eggs in figs as its hosts. The silkworm *B. more*, which mainly eats mulberry leaves, had only 29 lipase genes in total. The western honeybee *A. mellifera*, which lives in a highly standardized caste environment and develops by consuming a relatively specialized diet, had the lowest number of lipase genes (26). On the contrary, the omnivorous insects generally had a relatively large number of lipase genes (over 50): 62 in the fire ant *S. invicta*, 56 in the fruit fly *D. melanogaster*, and 54 in the red flour beetle *T. castaneum*.

Additionally, it seems that the lipase numbers in the lipase3 family among parasitoid wasps are generally larger than in the other species, which suggests that parasitoid wasps process a relatively different lipid metabolism, utilization, and absorption due to their unique life cycle. All insect species in our comparison had only one hormone-sensitive gene but lacked the lipase2 family gene. 

### 3.2. Expression Patterns of Encoding Lipases in P. puparum

Using a total of ten RNA-seq libraries data of *P. puparum* (embryos, 2-day larvae, female pupae, male pupae, female adults, male adults, venom glands, female carcasses, ovaries, and larval salivary glands), we calculated FPKM of each putative encoding lipase in each sample (Supplementary Materials Table S4) to represent its relative expression level. FPKM values of all predicted lipase genes ranged from 0 to 2022.12. Two heatmaps were constructed based on these values to display the lipase expression patterns across different developmental stages and tissues, respectively. 

**Figure 1:**
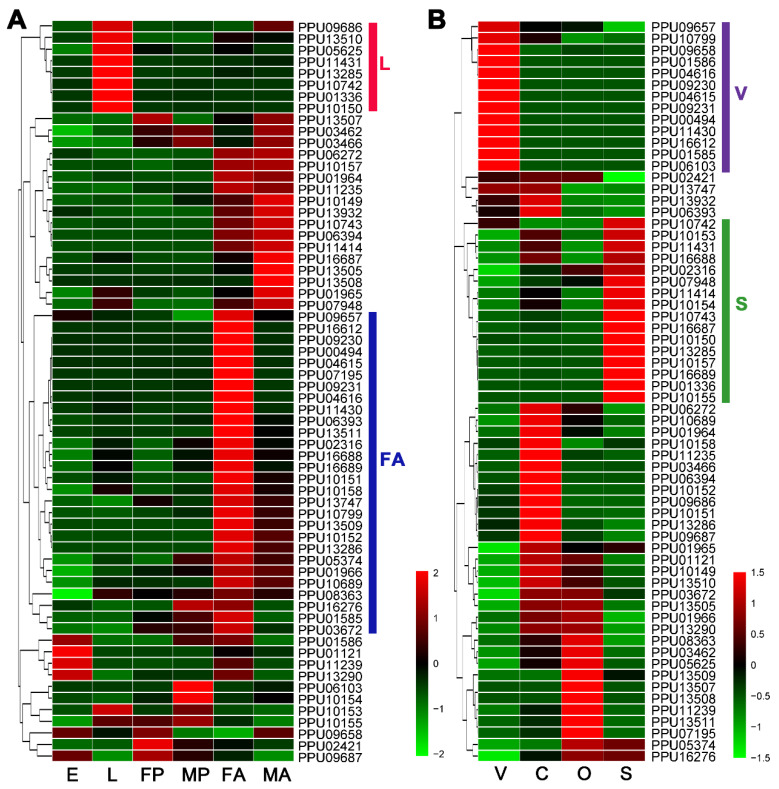
Heatmap analysis of *P. puparum* lipase genes across different developmental stages (**A**) and tissues (**B**). Log_2_FPKM values are indicted by a green–red bar where the red represents higher expression levels, the green represents the lower. Lipases are sorted into different groups according to their expression profiles. Colored bars at right represent the group names. E = eggs, L = larvae, FP = female pupae, MP = male pupae, FA = female adults, MA = male adults, V = female venom glands, C = carcass (female adults without venom glands), O = ovaries, S = larval salivary glands.

According to the heatmap based on the expression levels of developmental stages (Figure 1A), there were eight lipase genes with especially high expression at the larval stage (L group) and 28 lipase genes with particularly high expression in female adults (FA group). In addition, 13 lipase genes highly expressed particularly in venom glands were sorted into the V group, and 16 lipases with especially high-expression in larval salivary belonged to the S group (Figure 1B). 

**Figure 2:**
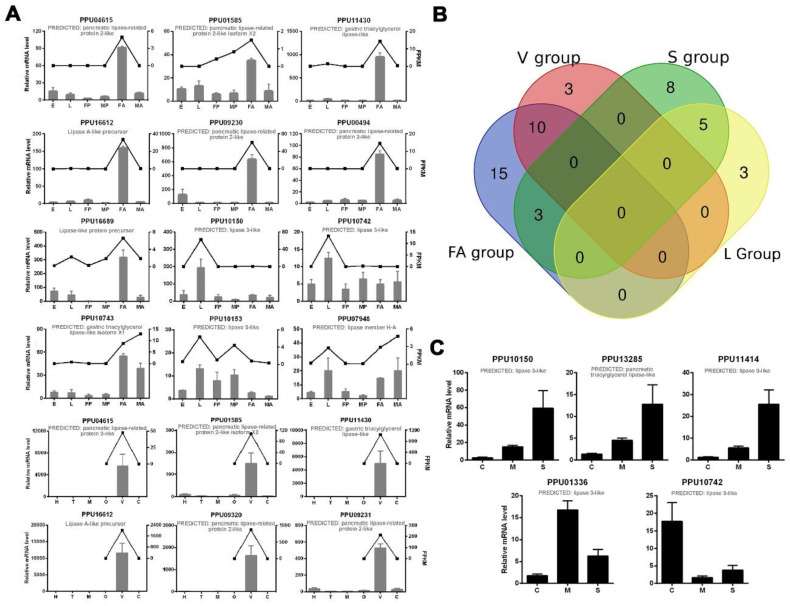
qPCR results of selected lipase genes in *P. puparum*. (**A**) Total RNA extracted from *P. puparum* across different developmental stages and female tissues were used to perform qPCR. Lines display the FPKM values in transcriptome analysis, corresponding to the Y-axis at right. qPCR results of the selected lipases were mostly in accord with the transcriptome data. E = eggs, L = larvae, FP = female pupae, MP = male pupae, FA = female adults, MA = male adults, H = heads, T = thoraxes, M = midguts, O = ovaries, V = female venom glands, C = carcass (female adults without venom glands). (**B**) Venn diagram of potential venom lipases and salivary lipases. The blue circle indicates the lipases with especially high expression in female adults (FA group), the red circle indicates lipases with especially high expression in venom glans (V group), the green circle indicates lipases with especially high expression in larval salivary glands (S group), and the yellow circle indicates lipases with especially high expression at the larvae stage (L group). (**C**). Total RNA was extracted from the larval tissues of *P. puparum*. C = carcass, M = midguts, S = salivary glands; 18s rRNA of *P. puparum* was used as a housekeeping gene. Error bars represent the standard errors of the mean from three biological replicates.

A number of predicted lipase genes of *P. puparum* were selected for qPCR analysis, and the results were mostly consistent with our transcriptomics analysis (Figure 2A). For further strict screening of venom-specific and salivary-specific lipases in *P. puparum*, a Venn diagram was drawn (Figure 2B). Ten lipases showed high-expression both at the female adult stage and in the venom gland tissue. Among them, two (PPU10799 and PPU09657) with a Log_2_ (venom-gland FPKM/carcass FPKM) value less than one were no longer considered to be putative venom-specific lipases in *P. puparum*. In the same way, five lipases had high-expression at the larvae stage and in the larval salivary gland tissue. We finally determined four of them as putative salivary-specific lipases with one exception, namely PPU10742. Although it was highly expressed in particular in the larval salivary gland, its FPKM was low at 0.5173. All the rest of the lipase genes in the S group had low expression levels (FPKM less than 10) at the larvae stage but high expression levels at the female adult stage. They were thus not considered as salivary-specific lipases in *P. puparum*.

To investigate the transcription levels of lipases in other larval tissues of *P. puparum*, we also performed qPCR for those putative salivary lipases using larval carcass, midgut, and salivary glands. Our results showed that three lipase genes were particularly highly expressed in larval salivary glands, while PPU01336 has a higher expression level in the midgut (Figure 2C). However, this lipase was not removed from putative salivary lipases. In *P. puparum* larvae, the salivary gland tightly adheres to midguts and thus the midgut samples usually mixed with the unremoved salivary gland. The higher expression level in midgut suggested that PPU01336 has high expression in midgut as well, but the comparison of expression between the salivary gland and midguts was still unclear. For further analysis, the positions of the signal peptides of those certain lipases were predicted, and their peptide-spectrum matches (PSMs) acquired from *P. puparum* venom proteome analysis [11] were displayed in Table 2. Nearly all putative venom lipases had at least two peptide-spectrum matches against the venom proteome except for PPU04615, with the lowest FPKM in the venom gland compared to others. 

While 75% (6/8) of putative venom lipases were predicted as pancreatic lipase-relative protein 2-like (PLRP2-like), no predicted PLRP2-like was found among putative salivary lipases. Half of the putative salivary lipases were identified as lipase3-like. For mammals, PLRP2 usually presents at the suckling–weaning transition to digest breast milk fats and carry through adulthood, participating in the cytotoxic activity of T-cells [43]. For endoparasitoids *P. puparum*, we suggest that some PLRP2-like proteins in venom probably contribute to the regulation of lipid metabolism in hosts and benefit the early development of endoparasitoid young. Subsequently, the larval salivary lipases may take over the duty of regulating the host lipid environment. The venom and salivary lipases may play different roles at different developmental stages of wasps.

### 3.3. Incomplete Catalytic Triads in Predicted Lipases of P. puparum

Enzymes in numerous families, including the α/β hydrolase fold superfamily, serine proteases, and brain acetylhydrolase, require a catalytic triad to drive the catalytic mechanisms [2]. For the neutral and acid lipases in mammals, a catalytic triad usually consists of Ser–Asp–His residues and produces a charge relay system to achieve the reaction mechanisms [44,45]. While many substitutions at these activity sites could lead to a non-functional enzyme, a minority could maintain the enzyme functions [35,46]. The frequent replacements occurred as Cys or Thr instead of Ser, Glu or His instead of Asp, and Lys instead of His [35].

**Figure 3:**
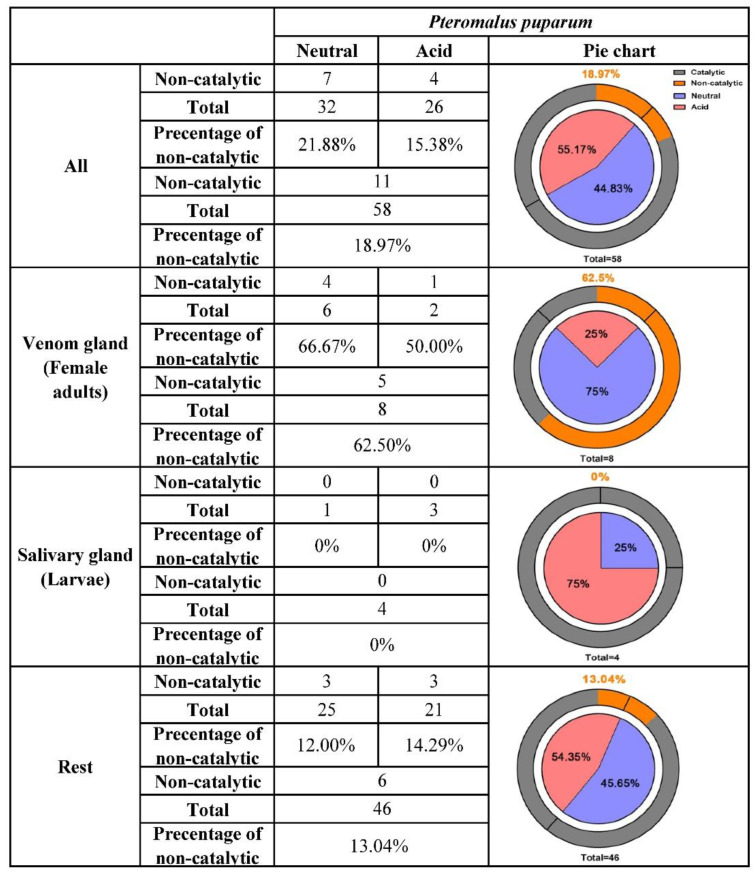
Summary of the neutral and acid lipase characteristics and their distribution in *P. puparum*. For the pie charts in the right columns, the blue scale in the inner circle represented the percentage of neutral lipases while the pink scale represented the percentage of acid lipases. Lipases without catalytic activity were displayed in the orange scale of the outer circle and its percentage was shown at the top of each pie chart.

In insects, an average of 10% neutral and acid lipases have generally lost the motif critical for catalytic function [36]. However, in *P. puparum*, this percentage was high at 18.97% (Figure 3). Eleven out of 58 putative neutral and acid lipases were found without a consensus catalytic triad. Interestingly, 72.73% of them (8 out of 11) with an inconsistent triad, belonging to the V group mentioned above, had especially high expression in the venom glands. The remaining three were highly expressed in the ovaries. It seems that all lipases without a typical catalytic triad were likely responsible for the development of wasp eggs. Focusing on the putative venom-specific lipases from the strict selection, we observed that 62.5% of them had incomplete catalytic triads. Most of them lost or altered a residue at His positions (including PPU00494, PPU01585, and PPU04615), while PPU04616 lost a residue at the Ser position. The only putative venom lipase gene with a conservative substitution (Glu) in place of Asp was PPU16612, which had the highest expression in venom glands among all lipases. This conservative replacement was not uncommon and had been found in two closely related lipases from mosquito salivary as well [47]. Among the putative salivary lipases, none was with replaced residues at the activity sites. The percentage of lipases with incomplete catalytic triads decreased to 13.04% when putative venom and salivary lipases were not considered.

Additionally, the percentages of neutral and acid lipases were likely polarized in the venom glands and salivary glands of *P. puparum*. A total of 32 neutral and 26 acid lipases were annotated in the *P. puparum* genome, occupying 55.17% and 44.83%, respectively. Among the putative venom lipases, 75% were neutral and 25% were acid. Neutral (25%) and acid lipases (75%) were found among the putative salivary lipases in *P. puparum*. While the putative venom and salivary lipases were not considered, the percentages of neutral and acid lipases were similar to the original levels (54.35% against 45.65%). In brief, many putative venom lipases are neutral while many putative salivary lipases are acid.

### 3.4. Identification of β9 Loop and Lids of Lipases in P. puparum

A β9 loop and a lid are two substantial features in mammalian neutral lipases, usually covering the active site (the catalytic triad) of lipases and involved in substrate recognition [48,49]. Although the minimum lengths of loops and lids required to generate functions are still unclear [49], many pieces of evidence have indicated the lipases with smaller numbers of residues in β9 loops or lids had weak TAG hydrolytic activity [50]. For a phosphatidylserine phospholipase A1 with both a short β9 loop (13 amino acids) and a small lid (12 amino acids), no TAG hydrolytic activity was detected in [51].

We constructed an alignment using serval recognized neutral lipase genes of holometabolous insects and all putative neutral lipase genes in *P. puparum* (Supplementary Materials Figure S1). As is known, the β9 loop with > 15 residues in length is essential for the TAG hydrolytic activity of lipases in insects [36]. The length of the β9 loop in neutral lipases of *P. puparum* ranged from 15 to 23 residues, except a quite short sequence PPU01585 with neither β9 loop nor lid (Table 3). Nearly all putative venom lipases had relative long β9 loops (19 to 21 residues), and the only putative neutral salivary lipase had a similar size β9 loop with 20-residue length. For the lids, at least 18 residues are usually required to present the normal TAG hydrolytic activity [36]. Over half of the predicted neutral lipase in *P. puparum* had lids smaller than 18 residues, as well as most neutral lipases with incomplete catalytic triads, which were highly expressed in venom glands (V group). It suggests that they may have weak TAG hydrolytic activity. Coincidently, the long β9 loops and shortened lids have been also found in the venom lipases from some parasitoid wasps. The lipase *DelePLA* from the endoparasitoid wasp *Diversinervus elegans* has 18 residues in β9 loop and 13 residues in the lid domain [17]. *c6971* from *Ooencyrtus telenomicida* has 18 and 16 residues in its β9 loop and lid domain, respectively [12]. For some social wasps and ants, their venom-associated phospholipase A1s (PLA1s) have been observed with both shorten β9 loops and small lid domains, indicating that these lipases lack TAG hydrolytic activity either [52,53,54].

Among 125 neutral lipases from 19 insect species analyzed by Horne [36], only 8 have both β9 loops greater than 15 and lids greater than 18 residues in length, meeting the consensus requirement for TAG activity in the mammalian enzymes. However, many more neutral lipases with predicted TAG hydrolytic activity were found in *P. puparum*. There were 14 neutral lipases meeting the β9 loop and lid criteria in *P. puparum*, including venom lipases PPU09230 and PPU09231. These two lipases had only 205 and 288 amino acids in length, but the longest lids among all neutral lipases, with 45 and 49 residues, respectively. They were both identified with a conserved catalytic triad.

### 3.5. Phylogenetic Analysis of Lipases in P. puparum

**Figure 4:**
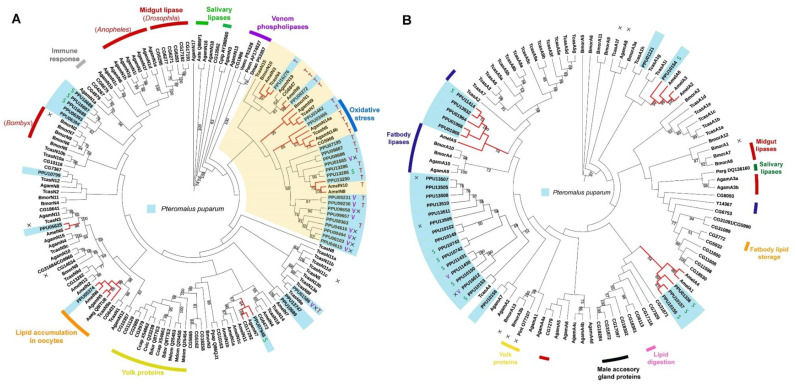
Phylogenetic trees of lipase proteins from *P. puparum* and other recognized insect lipases. (**A**) Neighbor-joining tree of neutral lipases. The yellow clade includes a cluster of lipases with predicted TAG hydrolytic activity. (**B**) Neighbor-joining tree of acid lipases. Lipases with especially high expression in venom glands and salivary glands are represented as letters V and S, respectively. Indications were used to display whether the proteins are catalytically inactive (×) or have putative TAG hydrolytic activity (T) predicted by the lengths of their respective β9 loops and lids. Branches in red represent the orthologues of *P. puparum* lipases. All predicated lipase proteins were displayed in a light blue box. Only bootstrap values (from 1000 replicates) over 50 were displayed as text. The functions or predicted functions of the lipases were presented at the outer circle in different colors.

Two phylogenetic trees were constructed using the predicted neutral and acid lipases of *P. puparum*. According to the neutral lipase phylogeny (Figure 4A), PPU05374 is closed to a neutral lipase from the yellow fever mosquito *Aedes aegypti* (Aaeg_ Q8ITU8AF303984) in a single phylogenetic clade. Aaeg_Q8ITU8 AF303984 has been deposited in the NCBI database with an annotated function as lipid accumulation in oocytes. In this clade, orthologues were also found in numerous insect genomes including *A. mellifera* (AmelN6), *A. gambiae* (AgamN5)*,* and *T. castaneum* (TcasN9a). Moreover, a neutral lipase of *P. puparum* PPU03466, clustered with *D. melanogaster* CG5966, a *T. castaneum* lipase (TcasN6), and two *A. gambiae* lipases (AgamN14a and AgamN14b) were found. While the expression of CG5966 increased under oxidative stress [55], the upregulation of TcasN6 [56] and AgamN14b [57] was observed during an immunological challenge. They were thus supposed to be responsible for membrane damage and new membrane construction. It indicates that PPU03466 in this cluster probably has a similar response. In the plasma membrane of *M. sexta* ovarian follicles, a 100 kDa membrane protein was found with DAG hydrolyze activity in hemolymph lipophorin [58]. PPU06272 and CG6847 were the only lipases with molecular weight >100 kDa in *P. puparum* and *D. melanogaster* genomes, respectively. CG6847 has been predicted as a midgut intracellular TAG lipase or a lipoprotein lipase involved in TAG mobilization from hemolymphatic lipophorin [36]. They were closely located in a conserved phylogenetic clade with AmelN9 and AgamN19, suggesting that they likely have a conserved function related to DAG hydrolysis. Besides, several orthologues were also found in our phylogenetic analysis. PPU05625 is the ortholog of AmelN5, PPU07948 is closed to AgamN7 and CG17292, and PPU03462 has orthologues such as BmorN1, AgamN9, and TcasN7. As orthologues, they probably have a similar function, although most of their functions are still unclarified. 

Interestingly, predicted lipase genes with especially high expression in venom glands (V group) clustered except for PPU01585 and PPU01586. Furthermore, nearly all those lipases with incomplete catalytic triads were located in a conserved clade. PPU08363 is the only lipase with a complete catalytic triad in this clade but has low expression levels across all different developmental stages and tissues. However, this non-catalytic clade was located far from the yolk protein clade whose lipases are generally with incomplete catalytic triads. Additionally, most neutral lipases with predicted TAG activity were observed in the yellow clade. A set of paralogs with predicted TAG hydrolytic activity likely evolved novel functions by gene duplications within the *P. puparum* genome.

In the acid lipase phylogenetic tree (Figure 4B), five *P. puparum* lipases formed an orthologous group with a fatbody lipase in *A. mellifera* (AmelA5). Three acid lipases with high expression levels in *P. puparum* larval salivary glands (PPU01336, PPU10157, and PPU10155) were identified as orthologs of acid lipases from *A. mellifera* (AmelA1 and AmelA4). PPU10154 was located in a conserved clade with *A. mellifera* lipase AmelA6 whose relevant ESTs (BI505714 and BI509544) were isolated from *A. mellifera* brain tissue [59]. Intriguingly, two putative acid venom lipases of *P. puparum* PPU11430 and PPU16612 were located in a closed relationship with PPU11431 and PPU10150, which were the putative salivary lipases of *P. puparum*. This suggests that those four acid lipases probably have a similar function in regulating the host lipid metabolism post-parasitization. 

## 4. Discussion

A total of 64 predicted lipase genes were identified in the *P. puparum* genome. They belong to five lipase families, including neutral, acid, lipase3, GDSL, and hormone-sensitive. To compare the numbers of lipases among different parasitoid wasp species, we also examined eight parasitoid wasp genomes to identify their lipases. Our results showed that *P. puparum* had the greatest number of lipases among all investigated insects. Based on data from ten RNA-seq libraries of *P. puparum*, the expression profiles of all predicted lipases were further analyzed. While 13 lipases had especially high expression in venom glands, 16 lipases had especially high expression in salivary glands. They were sorted into the V group and S group, respectively. Combining with their expression profiles at different developmental stages, we finally determined eight putative venom lipase genes and four putative salivary lipase genes. All 12 lipases were considered as key components participating in regulating the host lipid metabolism post-parasitized.

Lipases always have an α/β hydrolase fold and the helical portion known as a lid to cover the active site [60]. Once a lipase contacts lipid under suitable conditions, the active site opens and hydrolysis begins. For most lipases, the amino acid residues at this active site are Ser, Asp, and His. They consist of a classic catalytic triad and represent the catalytic activity of lipases [61,62]. Interestingly, the percentage of non-catalytic lipases in *P. puparum* was higher than the average (10%) in other insect species. Many lipases in venom glands of *P. puparum* were predicted as non-catalytic lipases. Some non-catalytic lipases were found with high expression in ovaries. It seems that those non-catalytic lipases were related to the development of wasp eggs. During the embryonic development, yolk proteins could function as some non-catalytic roles in lipid binding, lipid transport, and energy storage [63]. Numerous non-catalytic neutral lipases have been used as yolk proteins in some higher Diptera and acid lipases in Lepidoptera [64]. In some insect species, vitellogenins (the precursors of yolk proteins) have been replaced with non-catalytic lipase-like proteins. Since functional vitellogenin has been found in Hymenoptera [65], few lipase-like proteins performing a yolk protein function have been investigated within this order [22]. In *P. puparum*, non-catalytic lipases were mostly neutral and highly expressed in venom glands in particular. We assumed that while *P. puparum* injects venom along with eggs into hosts, these non-catalytic venom lipases probably act as yolk proteins, binding host lipids to deliver energy for egg development in parasitized hosts. Nevertheless, more experiments have to be performed to validate our hypothesis.

Nevertheless, venom lipases with an incomplete catalytic triad were not widely found in parasitoid wasps. Among all recognized venom lipases as reported, an acid lipase NV21220 from *N. vitripennis* (Pteromalidae) was the only one with an incomplete catalytic triad (Ser137–Glu401–Asp434). Two well-characterized venom lipases in *O. telenomicida* (*c6971*) and *D. elegans* (*DelePLA*) have been reported with a high consensus catalytic triad previously [12,17]. In other words, the venom proteins with incomplete catalytic triads may not widely exist in parasitoid wasps. They frequently present in *P. puparum* in particular, which has the largest number of predicted lipase genes among all eight parasitoid wasps in our analysis. It suggests that different parasitoid wasps probably use a variety of strategies to regulate and utilize host lipids. 

Two important features responsible for the significant TAG hydrolytic activity of lipases are a β9 loop and the lid, which cover the active site. According to our prediction based on the lengths of β9 loops and the lids, nearly all of non-catalytic neutral lipases of *P. puparum* have short lids indicating weak TAG hydrolytic activity. It seems that the incomplete catalytic triads are interconnected with the small lids. However, some reported venom-associated lipases in other wasps generally have both classic catalytic triads and small lids. Moreover, *P. puparum* has a greater number of lipases with predicted TAG hydrolytic activity, compared to those reported lipases from other insect species. This number in *P. puparum* is even greater than the total number in all Holometabolous insects analyzed by Horne et al. [36]. Based on our analysis of the phylogenetic dendrogram, most of these lipases in *P. puparum* are in-paralogues and likely have evolved novel functions. They clustered into a sister clade of the venom lipases clade in *P. puparum*, suggesting that they are probably responsible for the unique life cycle of *P. puparum* as well.

## 5. Conclusions

It is generally believed that many parasitoid wasps have lost the capability to synthesize lipids themselves and have to obtain lipids from hosts during development. Our genome-scale identification and expression analysis of *P. puparum* lipases partially contribute to the understanding of lipases in parasitoid wasps, which is essential to figure out how parasitoid wasps utilize host lipids for development. The frequent observation of non-catalytic lipases in *P. puparum* venom suggests that an uncommon function of lipases has been evolved in parasitoid wasps. We speculate that future studies may need to focus on various molecular mechanisms of lipases inside parasitoid wasp venom. Although the TAG hydrolytic activity of lipases was not experimentally validated, it was predicted by analyzing sequences’ characteristics, and a general feature in venom lipases was discussed. We look forward to forthcoming insights in the insect lipase database and predict that an increasing number of insect lipases could be probably developed and explored in different application fields.

## Figures and Tables

**Table 1:** Numbers of predicted lipase genes from each lipase family of selected insect species.

Species	Neutral	Acid	Lipase2	Lipase3	GDSL	Hormone Sensitive	Total
*Pteromalus puparum*	32	26	0	3	2	1	64
*Nasonia vitripennis*	30	27	0	2	2	1	62
*Solenopsis invicta*	28	25	0	2	6	1	62
*Trichogramma pretiosum*	30	24	0	2	2	1	59
*Drosophila melanogaster* ^1^	31	21	0	1	2	1	56
*Tribolium castaneum* ^1^	25	25	0	1	2	1	54
*Anopheles gambiae* ^1^	28	14	0	1	7	1	51
*Diachasma alloeum*	18	22	0	3	4	1	48
*Copidosoma floridanum*	24	17	0	2	2	1	46
*Fopius arisanus*	14	19	0	2	2	1	38
*Ceratosolen solmsi*	16	11	0	2	1	1	31
*Bombyx mori* ^1^	11	14	0	1	2	1	29
*Orussus abietinus*	18	6	0	2	1	1	28
*Apis mellifera* ^1^	14	4	0	1	6	1	26

^1^ Data source: Horne et al. [36].

**Table 2:** Information of the lipases with especially high expression in venom glands and larval salivary glands of *P. puparum*.

Tissue	Gene ID	Position of Signal Peptide	PSMs	Tissue_FPKM	Log2(Vg_FPKM/Ca_FPKM)	Description	Accession
Venom gland	PPU04615	1–19	N	47.8084	∞	PREDICTED: pancreatic lipase-related protein 2-like	XP_016836798.1
	PPU01585	1–16	7	107.410	∞	PREDICTED: pancreatic lipase-related protein 2-like isoform X2	XP_016845667.1
	PPU09230	N	13	868.856	10.60339576	PREDICTED: pancreatic lipase-related protein 2-like	XP_016838373.1
	PPU11430	1–17	22	1041.80	10.1179628	PREDICTED: gastric triacylglycerol lipase-like	XP_016840181.1
	PPU16612	1–19	51	2022.12	9.693282524	lipase A-like precursor	NP_001154991.1
	PPU00494	N	5	1526.20	9.52790379	PREDICTED: pancreatic lipase-related protein 2-like	XP_008215592.1
	PPU04616	1–18	2	265.566	8.762219154	PREDICTED: pancreatic lipase-related protein 2-like	XP_016836798.1
	PPU09231	1–22	12	211.041	8.503220789	PREDICTED: pancreatic lipase-related protein 2-like	XP_016838373.1
	PPU10799	N	N	10.1819	0.929008269	PREDICTED: pancreatic lipase-related protein 2-like	XP_008217694.1
	PPU09657	1–21	N	37.3270	0.540844581	PREDICTED: pancreatic lipase-related protein 2-like isoform X1	XP_003425032.1
Larval salivary gland	PPU01336	1–20	-	113.132	-	PREDICTED: lipase3-like	XP_016841509.1
	PPU10150	1–20	-	24.8997	-	PREDICTED: lipase3-like	XP_008216710.1
	PPU11431	N	-	5.76564	-	PREDICTED: gastric triacylglycerol lipase-like	XP_016840181.1
	PPU10742	1–20	-	0.517292	-	PREDICTED: lipase3-like	XP_015112844.1
	PPU13285	1–23	-	6.65252	-	PREDICTED: pancreatic triacylglycerol lipase-like	XP_016844594.1

N indicates no predicted signal peptide was found; ∞ indicates the infinite value from division by zero.

**Table 3:** Predicted activity site residues, β9 loop and lid domains of *P. puparum* neutral lipases.

Tracking_ID	Predicted Active Site Residues	β9 Loop	Length	Lid	Length
PPU01585	S34D62-	-	0	-	0
PPU00494	S16D44-	HTQGGKRDNNKAFGLNALLG	20	-	0
PPU16687	S158D182H244	HTNVDNCGMTYQVGH	15	CNSNRC	6
PPU16688	S163D187H249	HTDIQECGLKDQIGH	15	CEKHKC	6
PPU16689	S191D215H280	HTNAGLLGYLSAIGK	15	CSIDIGGSC	9
PPU13747	S187D211H280	HTCAGTVGFVRPIGH	15	CPVLMTQYC	9
PPU06394	S164D188H254	HTNGGNLGIRYPLGH	15	CGADLIGSC	9
PPU04615	S177D206-	HTQTGNGEKISGFGLQKPSGH	21	CEIKSDGYV	9
PPU10689	S171D195H265	HTSGTAFGFLAAIGH	15	CNFAPTNTYC	10
PPU05374	S170D194H259	HTNAGYYGELGKVGH	15	CENRPNHELC	10
PPU06393	S80D104H172	HTNSNYYGLPEPRGH	15	CADEPNQKYYC	11
PPU10799	S235D260H329	HSCGGVLGFLQPLGH	15	CCCVPELIEAC	11
PPU05625	S172D197H265	HTGAGILGQWGPNGH	15	CATASLLQTLSC	12
PPU07948	S164D188H258	HTDGGIYGAYEPTGS	15	CFLFGVPLSPRGL	13
PPU09658	S25D53I128	HTQTGTGGSVDGLGLKESIGH	21	CVRKKVGDDYLKN	13
PPU06103	S129D157Q232	HTQTGNDEDISGIGVQERSGH	21	CETKHMTIQKMLC	13
PPU09657	S176D204H279	HTQTGTGGSVDGLGLKESIGH	21	CVSKTLKWDNMIC	13
PPU04616	-D203H281	HTQTGHGNGINAFGLEAPVGH	21	CEAKSIFYTEVNKMIC	16
PPU08363	S174D203H283	HTQTGNGNGINGLGLQESIGH	21	CERVSSIIHTTRIQKMIC	18
PPU01586	-	HTDAVRTKNDEFGIRDPIGH	20	CDRRKRSFVTCWAMVAAIILEL	22
PPU13290	S266D295H379	HTNGQFLKKLGLGLPQPIGH	20	CELTSFTIPVLSIPREAINKAIC	23
PPU13286	S111D144H229	HTNAQNIMILGFGLPTQLGH	20	CAKIDTSFWDFLLLPVNIVKSAI	23
PPU13285	S263D296H381	HTNARQIYFLGLGLPEQLGF	20	CSDIDTSIWSFLLLPKTIIQESIC	24
PPU16276	S193D220H308	HTDGSVDFADGFGLLKPIGH	20	CKDVKNSVVVSHLNEDSLDIHIAC	24
PPU07195	S208D237H322	HTNAKGSLTEGLSLFKPIGH	20	CSESNFILPDSIKLPKRIIDEAVC	24
PPU09686	S464D493H578	HTNGRVLSKLGLGLPYPVGH	20	CILSETSLWRYLPLPIQKISETIC	24
PPU09687	S229D258H343	HTNGRILKKLGLGLPYPLGH	20	CILAKSSIWKYLPLPIEKIKKTIC	24
PPU06272	S218D241H326	HSNGEQLILGGLGSWQPMGD	20	CSNLFVGAVSDIIWSSPVEGRSLC	24
PPU03466	S212D240H327	HTDCSPFISGGLGISQPVAH	20	CNEGVFNSITLEKGSFFRGIKRFLGC	26
PPU03462	S231D255H349	HTDGKSIFFLGLPGYGMSQPCGH	23	CTDLSETTPSLPLTLIREGLEEASRVLVAC	30
PPU09230	S71D97H202	HTNSNPSGDTFGLYEPLGH	19	CSSNRVARTFSKDSVFIKCFSELFLGMDLSQLYGNIKSSRVELAK	45
PPU09231	S71D98H208	HTNSDPNRSTLGLYERLGH	19	CNNNQARTFSLWDAWEAIKNCTLIYFAGDNYSDLASEILTNLLTHSMVC	49

Background in purple: venom lipases; Background in green: salivary lipases; Letters in red: incomplete catalytic triads.

## Supplementary Materials

The following are available online at https://www.mdpi.com/2075-4450/11/4/227/s1. Supplementary file 1. Seven well-characterized lipase protein sequences from six lipase families in *D. melanogaster* genome. They were used as probes to identify lipase genes in *P. puparum*. Figure S1: Alignments of eight recognized insect lipase sequences and all putative neutral lipases annotated from *P. puparum*. Red boxes indicate the predicated β9 loop domains and lid domains. The numbers at the right show the position of the sequence. Table S1: The lipases identified in the venom of various parasitoid wasp species. Table S2: Primers of the selected lipase sequences for qPCR. Table S3: The information of all identified lipases of *P. puparum*. Table S4: FPKM values of lipases from different libraries of *P. puparum*.
