# Supplementary material for: Insight into the Functional Diversification of Lipases in the Endoparasitoid Pteromalus puparum (Hymenoptera: Pteromalidae) by Genome-scale Annotation and Expression Analysis
Source: Insects. 2020 Apr 5;11(4):227. doi: 10.3390/insects11040227 (PMC7240578; doi:10.3390/insects11040227)
Supplement: Supplementary file 1 [file insects-11-00227-s001.zip › Supply/Supplementary file 1.docx]

>CG11029-PA GDSL

MASITYALCLCSLFILFSPLVSANRRVRRQNRSDRLLADIGVRAQSYDPRVPENGIQQYTDIDQDLRHLFLNTRQTTLKWALNNIEALSSRGRREGKLQAPVSKKVPFLCPTNNTRSPSPPTSIEHLRPGDIDIIAAFGDSLSAGNGILSNNAIDMINEFRGLTFSGGGLANWRRFVTLPNILKIFNPKLYGFAVSNSLVINHRSSRLNIAEPMIMSRDLPFQARVLIDLLRRDRHVDMKRHWKLLTVYVGNNDICSDLCHWDTPQSFLDQHARDLRQAFRLLRDHVPRLLINLIVVPNIPLVLSTMTKVPLQCFVVHRVGCHCLINDRLNRTEFNERMDTLTRWQQLDMEIARLPEFRRQDFAIVAHPMLTKLTAPTLPDGSTDWRFFSHDCFHFSQRGHAIISNLLWNSMLLPDDQKPRPSVVPELFERVVCPTAEQPYLVVRPS

>CG33174-PA-lipase3

MPGLVVFRRRWSVGSDDLVVPGAFLLTIHFICFVIVSVSLVIFEYNTRILSVKLLFYHLIGYLLILFFSICVEIGICVISMRGSILDAEARTSINIWIYLKSLVILFDIAWLAVGSVWLGHYYTTAPIDDPKKVYIAIIICNWALVVITLITIWCTFDAAGRSWVKMKKYQRSMRETESRFNYKRSNSMNRNWRQRKVMRAYQDSWDHRCRLLFCCMGSSERNRNSFTDIARLLSDFFRELDVVPSDVVAGLVLLRKFQRLEREAIVRQRKNGTYEFLSGVPITERTQFLALNDAKNYDFFQTVIHYMYFAQGAYGWPMYVIINRSKMWHLVPELKCFGCCCGTSDDTEVIQDNCCLCNYAALKKTLQLGDIDIVYATYHVDVGETPFFVAIDYTHRAVVISIRGTLSMKDILTDLNAEGEVLPLQPPRDDWLGHKGMVQAAIYIRNKLQEENLIERALQRNPDRQTHTFDLVLVGHSLGAGTAAILAILLKPEHPTLQCFSYSPPGGLLSMPAVEYSKSFITSVVLGKDVVPRIGLNQMEALRADLINAIQRSVDPKWKTISCSVICCGCGPEPTSVVNMSGQDTHINQYQEERGTARSTSAHPTDSSIALTLHQPLYPPGRIIHIVRHHPKPDEQKYDSGWR

>CG11055-PA-HSL

MIDAASAERASPQFIALFNDNLKLGHEDDGPQAVNGKDTHQHVPADLEATYGTLYAACQDHAAFFARDHTEFGQRLHAAHIAWQDFIVLANRLVQQIDAFAHEYDFDEQTPGNGYRSFIYVTNACIAHGISICQQLTATRSTIFFRKKFYMKEVEACSQLLSSLCTCLQYLLILRQWSASTGDLFACGNHTAEQLFELGDTINQYCFYGRCLGFQYGDSIRGVLRFLGISMASYSESYYSQEGDGPIVKTTRSLWTSGKYLMNPELRARRIVNISQNAKIDFCKSFWFLAESEMMHKLPSIVGSSIKVNRLIELPAEPLK

LPRRKNFKASDNLSSDVNQNQGDGDFVEIPVPTAHLGPGLPVSVRLLSARRRSGMLGEGRYRGWHKPIPPSPSILFHCHGGGFVAQSSKSHELYLRDWAVALDCPILSVDYSLAPEAPFPRALQEVYYAYCWLLNNTELLGTTAERVVCAGDSAGANLSIGVALKCIEQGVRVPDGLFLAYCPTLVSFVPSPARLLCLMDPLLPFGFMMRCLRAYAAPAQETLQQNAKQVEDAAQIRNVPKSNVGSLNSSRRTSMARSPLSPLEASMNPDDESSDTFASASASYHSQTVERTDLPHTEGDNSSCVSFEDDSQPIVHYPIEISADPPKDTASAAYIDNFLDKYLIDTATMEVTEETAPEAVQAQANGHAKISSDDDILVETGRDLVAIDTLQGRLQEAVNNITNTLTRCTQSYEIHGSNVMAQQDVRNMDALIARSPSEEFAFDVPKDPFLSPYWASDEWLSQLPETKILTLNMDPCLDDCVMFAKKLKRLGRQVDLEILEGLPHGFLNFTMLSNEAMEGSKKCIKSLQTLLQTDSKTKNIDKANSMDEEESSSPSASLAAS

>NP_496693 lipase2

MSSAVSSVAADSSRSNSTYGMAAARTDGDGSYGGGSHVGNSATSKRVHGATNTAASARDYMSHGWSATVYGTTYGDGGTCVDDYDVDGDSTSVTYVSVAGANRGSGCVATTSATCNRVNGWCASRKDNTHYGSKVSYGRNDDKVMSNCWSRSSRNGADGMDNAGNHDSGTVTHS

>CG6283-Neutral

MKVFFVLAALLAAVSALPIEERVNGENGWFIPKLDGSFEWMDMQDAEDLLANGAQMEGRISTNAVNFYVYTKSNPTDGKEIKAKSGSVEDSHFNKDHGTRFVIHGWTQRYSDDMNTRITKAWLSKGDYNVIVVDWARARSVDYASSVLAVPGAGGKVGEMIKYLHDHHGLDYDSLEVIGHSLGAHVAGYAGKTVGDKRVHTIVGLDPALPLFSYDKPAKRLSTDDAHYVESIQTNGGKLGFLKPIGKGAFYPNGGKSQPGCGLDATGSCSHARSVLYYAEAVTEDNFGSIKCHDYEDAVAKNCGSTYSSVRMGAITNAYMVEGDFYVPVNSEAPFGKIE

>CG6753-acid

MRRERVVLLCLALLGLQQVSRATLRQSREIIITDAVRRIQNDGYNVERHSVTTKDGYVLTLHRIPQVDPELGSLLRRPVVFLLSGLYASSDVWLLNGREDSLAYLLWRAGYDVWLGNNRGNIYCRKNMWRNTTEREFWDFSWHEMGVYDLPAQVDYVLRTTGQKAMHFVGISQGGTVFLVLNSMMPQYNAVFKSATLLAPVAYVSNTKSGLAKVIGPVLGTRNYVSKMLEGVEMFSTNKFFKKFLSMTCLENEKPLVCISRLWPAVGYDTRFLNKTLLPDLMANFPAGGSVKQLMHYFQGYVSTRFRQYDYGPERNWLHYQQLEPPEYALENVSTPVTVFFSENDYIVAPADIWRLLTRLPNVEAVYKVPWKRWNHFDFICGLGVREYIFDNIVLSMNRYEQRRR

>CG11598-acid

MSYKAFVFFCLYIDLAKGLITSEIIASHNYPVEVHTVLTRDGYLLDAFRIPGSKFCQQSGPKPAVLFQHGMSASSDVFLLNGPQDSLAFMLADACFDVWLSNSRGTRYSRRHVSLDPSDEAFWRFSWHEIGTEDVAAFIDYILDTTKQRALHFLGHSQGCTTLVVLLSMRPEYNKLVKTAVLLAPAVFMRHTSTLSQTVFRSFIMAMPDKEFMYHNGVLNKLLSNVCGLFVARVFCTTFFLISNGKISKHLNTSVIPLIAATLPAGVSSRQPKHFIQLTDSGKFRPFDFGILRNLINYKSLEPPDYTLSNVRPLTPVHIFYSDDDSSTAKEDIQNFAARVPEVVMHRISTPGWHHTDFVHSMTVADVINKPVIEIFRSFERLSSPTFL

>CG7279-acid

MRCSLRMQLLLLLGLCVFISRIQGQLIGGEEDEEDEEEEEEEEESVEDETPEDRLQRKNIKQDSTLSVDKLIAKYGYESEVHHVTTEDGYILTMHRIRKQGAPPFLLQHGLVDSSAGFVVMGPNVSLAYLLADHNYDVWLGNARGNRYSRNHTTLDPDESKFWDFSWHEIGMYDLPAMIDHVLKVTGFPKLHYAGHSQGCTSFFVMCSMRPAYNDKVVSMQALAPAVYAKETEDHPYIRAISLYFNSLVGSSIREMFNGEFRFLCRMTEETERLCIEAVFGIVGRNWNEFNRKMFPVILGHYPAGVAAKQVKHFIQIIKSGRFAPYSYSSNKNMQLYRDHLPPRYNLSLVTVPTFVYYSTNDLLCHPKDVESMCDDLGNVTGKYLVPQKEFNHMDFLWAIDVRKMLYRRMLQVLGKVPEGSPEEANRSRREIRGKFIRS
