# Supplementary material for: Insight into the Functional Diversification of Lipases in the Endoparasitoid Pteromalus puparum (Hymenoptera: Pteromalidae) by Genome-scale Annotation and Expression Analysis
Source: Insects. 2020 Apr 5;11(4):227. doi: 10.3390/insects11040227 (PMC7240578; doi:10.3390/insects11040227)
Supplement: Supplementary file 1 [file insects-11-00227-s001.zip › Supply/Table S1.docx]

| **Table S1. The lipases identified in the venom of various parasitoid wasp species.** | | |  |  |
| --- | --- | --- | --- | --- |
| **Species** | **Seq/Accession number** | **Description** | **Identification method** | **References** |
| ***Ooencyrtus telenomicida*** | c7435_g5 | PREDICTED: lipase 3-like | Tanscriptomics | Cusumano *et al.*, 2018 [12] |
|  | c7435_g4 | PREDICTED: lipase 3-like |  |  |
|  | c6971_g1 | PREDICTED: pancreatic lipase-related protein 2-like |  |  |
|  | c7740_g4 | PREDICTED: pancreatic lipase-related protein 2-like |  |  |
|  | c7740_g2 | PREDICTED: pancreatic triacylglycerol lipase-like |  |  |
| ***Toxoneuron nigriceps*** | Spot 78,85 | Phospholipase a2-like | Proteinomics | Laurino *et al*., 2016 [13] |
| ***Psyttalia lounsburyi*** | Pl_006057 | Esterase/lipase-like | Transcriptomics | Mathé-Hubert *et al*., 2016 [14] |
|  | Pl_009261 | Esterase/lipase-like | Transcriptomics & Proteinomics |  |
| ***Psyttalia concolor*** | Pc_002246 | Phospholipase A2 | Transcriptomics & Proteinomics |  |
| ***Nasonia vitripennis*** | hmm642184 | Lipase | Proteinomics | De Graaf *et al*., 2010 [15] |
|  | hmm589104 | Lipase-like venom protein |  |  |
|  | Nasvi2EG007615/NV21220 | Lipase | Transcriptomics | Sim and Wheeler, 2016 [16] |
|  | Nasvi2EG004342/NV21343 | Lipase-like |  |  |
| ***Diversinervus elegans*** | c5504_g1 | Phospholipase A1 | Proteinomics | Liu *et al.*, 2017 [17] |
| ***Chouioia cunea*** | unknown | Lipase | Proteinomics | Xin *et al.*, 2017 [18] |
|  | unknown | Phospholipase A1 |  |  |
| ***Chelonus inanitus*** | Ci-50 | Esterase/lipase-like | Proteinomics | Vincent *et al.*, 2010 [19] |
| ***Leptopilina heterotoma*** | 3H06 | PREDICTED: phospholipase B-like | Transcriptomics | Heavner *et al*., 2013 [20] |
|  | 1H07 | Lipase 3 |  |  |
| ***Leptopilina bboulardi* ISm** | lbm_CL29Contig1 | Lipase | Proteinomics | Colinet *et al.*, 2013 [21] |
|  | lbm_CL178Contig1 | PREDICTED: lipase 3-like |  |  |
| ***Leptopilina bboulardi* ISy** | lby_CL86Contig1 | Lipase |  |  |
| ***Microplitis demolitor*** | comp40703_c0 | Esterase/lipase-like | Transcriptomics | Burke *et al.*, 2014 [22] |
|  | comp38077_c0 | Phospholipase A2 |  |  |
| ***Pteromalus puparum*** | comp44819_c3 | Lipase A-like precursor | Transcriptomics & Proteinomics & Signal peptide prediction | Yan *et al.*, 2016 [10] |
|  | comp28596_c0 | Lipase-like venom protein precursor |  |  |
|  | comp41786_c2 | Lipase-like venom protein precursor |  |  |
|  | comp42555_c0 | PREDICTED: lipase member H-like |  |  |
|  | comp45112_c0 | PREDICTED: pancreatic lipase-related protein 2-like |  |  |
|  | comp28462_c0 | PREDICTED: pancreatic lipase-related protein 2-like |  |  |
|  | comp22275_c0 | PREDICTED: pancreatic lipase-related protein 2-like |  |  |
|  | comp36060_c0 | PREDICTED: pancreatic triacylglycerol lipase-like |  |  |
|  | comp22302_c0 | PREDICTED: hypothetical protein LOC100122136/region_name= Abhydro_lipase |  |  |
|  | comp44469_c0 | PREDICTED: esterase E4 |  |  |
