# Supplementary material for: Insight into the Functional Diversification of Lipases in the Endoparasitoid Pteromalus puparum (Hymenoptera: Pteromalidae) by Genome-scale Annotation and Expression Analysis
Source: Insects. 2020 Apr 5;11(4):227. doi: 10.3390/insects11040227 (PMC7240578; doi:10.3390/insects11040227)
Supplement: Supplementary file 1 [file insects-11-00227-s001.zip › Supply/Table S2.docx]

**Table S2. Primers of the selected lipase sequences for qPCR.**

| **Gene ID** | **sp** | **ap** |
| --- | --- | --- |
| PPU04615 | TAGATCCAGCGCAACCTTGT | CTGGTTGCATTACACCACCA |
| PPU01585 | CAGTCCTGAAAGTTGGGCAC | TTGGCTTCCTCGAAACATGG |
| PPU11430 | TCTTATACTCCCGCTGGCAC | GTATTGCGGTGGTGTCGTTT |
| PPU16612 | GCGCCACAATTTTGACGAAG | GATGTTCCCGCTGGCATATG |
| PPU09230 | AAGCGGTGACACTTTTGGTC | GCGAGCTCAACTCGAGAACT |
| PPU00494 | TTATCGGGCACAGTCTTGGT | AATGCGTTTAACCCAAAAGC |
| PPU16689 | ACCCAGCTCTTCCTCTGTTC | GGTGATGGACTCTGCGTAGT |
| PPU10150 | TCAGGGGAATTCGTGGTTCT | TTTTCCAGCAGCTTTAGGGC |
| PPU10742 | ACGGCAGAAGAGTACAAGCT | TAACTGTTACCTCGGGCGTT |
| PPU10743 | AGACGGCACTCCCACAAATA | TCACCTTTCGCGTACCAGAT |
| PPU10153 | TGAACACGAGGGGCAACTAT | TCGTGTTGACCTTGGCATTG |
| PPU07948 | TATCCCAAGAGTTACCGGCC | GGCACTCCAAACAAGAAGCA |
| PPU09231 | ACAGTTGATTGGTCGCACAC | ATATGAGCCCCGAGACTGTG |
| PPU10150 | TTTACTGCGCTTCCAGAGGT | CGGTGACGCTAACATACACG |
| PPU13285 | AAGGAAGCAACACGTGGAAC | GGCCAACAAAGTACAGCGAA |
| PPU11414 | AGGGTTTGACTCCGAGCATT | CGTAGTTAGGAGGTTCGGGA |
| PPU01336 | GGCTACATCCTCGAAGTCCA | GGTTGGCATGCTTACGAGAG |
