# Supplementary material for: Insight into the Functional Diversification of Lipases in the Endoparasitoid Pteromalus puparum (Hymenoptera: Pteromalidae) by Genome-scale Annotation and Expression Analysis
Source: Insects. 2020 Apr 5;11(4):227. doi: 10.3390/insects11040227 (PMC7240578; doi:10.3390/insects11040227)
Supplement: Supplementary file 1 [file insects-11-00227-s001.zip › Supply/Table S3.docx]

| **Table S3. The information of all identified lipases of *P. puparum*.** | | | | | | |  |  |  |
| --- | --- | --- | --- | --- | --- | --- | --- | --- | --- |
| **Gene ID** | **Predicted active site residues** | **Subfamily** | **Scaffold number** | **Start position** | **End position** | **Description** | **E-value** | **Identity Percent** | **Accession** |
| PPU00494 | S16D44- | Neutral | Scaffold_247 | 269968 | 270240 | PREDICTED: pancreatic lipase-related protein 2-like [*Nasonia vitripennis*] | 0 | 96% | XP_008215592.1 |
| PPU03462 | S231D255H349 | Neutral | Scaffold_1 | 2019198 | 2028391 | PREDICTED: pancreatic triacylglycerol lipase-like isoform X1 [*Nasonia vitripennis*] | 0 | 99% | XP_008212236.1 |
| PPU03466 | S212D240H327 | Neutral | Scaffold_1 | 2073019 | 2084156 | PREDICTED: pancreatic triacylglycerol lipase-like [*Nasonia vitripennis*] | 0 | 96% | XP_016844350.1 |
| PPU04615 | S177D206- | Neutral | Scaffold_0 | 13488855 | 13489948 | PREDICTED: pancreatic lipase-related protein 2-like [*Nasonia vitripennis*] | 3.00E-116 | 62% | XP_016836798.1 |
| PPU04616 | -D203H281 | Neutral | Scaffold_0 | 13491852 | 13493541 | PREDICTED: pancreatic lipase-related protein 2-like [*Nasonia vitripennis*] | 0 | 90% |  |
| PPU05374 | S170D194H259 | Neutral | Scaffold_2 | 1678875 | 1680871 | PREDICTED: phospholipase A1 [*Nasonia vitripennis*] | 0 | 98% | XP_001607221.2 |
| PPU05625 | S172D197H265 | Neutral | Scaffold_2 | 5182003 | 5188832 | PREDICTED: phospholipase A1 member A [*Nasonia vitripennis*] | 0 | 99% | XP_001603737.1 |
| PPU06103 | S129D157Q232 | Neutral | Scaffold_5 | 2992317 | 2993723 | PREDICTED: pancreatic lipase-related protein 2-like [*Nasonia vitripennis*] | 0 | 85% | XP_003425034.3 |
| PPU06272 | S218D241H326 | Neutral | Scaffold_5 | 5475870 | 5500445 | PREDICTED: uncharacterized protein LOC100114392 [Nasonia vitripennis] | 0 | 95% | XP_001599427.2 |
| PPU06393 | S80D104H172 | Neutral | Scaffold_4 | 573401 | 574917 | PREDICTED: pancreatic lipase-related protein 2-like [*Nasonia vitripennis*] | 6.00E-176 | 95% | XP_001608010.2 |
| PPU06394 | S164D188H254 | Neutral | Scaffold_4 | 577816 | 579472 | PREDICTED: phospholipase A1-like [*Nasonia vitripennis*] | 0 | 81% | XP_001608012.1 |
| PPU07195 | S208D237H322 | Neutral | Scaffold_7 | 3039075 | 3040869 | PREDICTED: pancreatic triacylglycerol lipase-like isoform X2 [*Nasonia vitripennis*] | 0 | 92% | XP_008204675.1 |
| PPU07948 | S164D188H258 | Neutral | Scaffold_9 | 3734825 | 3738763 | PREDICTED: lipase member H-A [*Nasonia vitripennis*] | 0 | 91% | XP_001599078.1 |
| PPU08363 | S174D203H283 | Neutral | Scaffold_8 | 3525338 | 3527012 | PREDICTED: pancreatic lipase-related protein 2-like isoform X1 [*Nasonia vitripennis*] | 0 | 90% | XP_003424474.1 |
| PPU09230 | S71D97H202 | Neutral | Scaffold_150 | 206795 | 208264 | PREDICTED: pancreatic lipase-related protein 2-like [*Nasonia vitripennis*] | 7.00E-120 | 87% | XP_016838373.1 |
| PPU09231 | S71D98H208 | Neutral | Scaffold_150 | 213866 | 215055 | PREDICTED: pancreatic lipase-related protein 2-like [*Nasonia vitripennis*] | 1.00E-66 | 47% |  |
| PPU09657 | S176D204H279 | Neutral | Scaffold_30 | 556949 | 559892 | PREDICTED: pancreatic lipase-related protein 2-like isoform X1 [*Nasonia vitripennis*] | 0 | 95% | XP_003425032.1 |
| PPU09658 | S25D53I128 | Neutral | Scaffold_30 | 562213 | 563241 | PREDICTED: pancreatic lipase-related protein 2-like isoform X2 [*Nasonia vitripennis*] | 8.00E-77 | 95% | XP_016841760.1 |
| PPU09686 | S464D493H578 | Neutral | Scaffold_30 | 1006532 | 1012529 | PREDICTED: pancreatic triacylglycerol lipase-like isoform X1 [*Nasonia vitripennis*] | 0 | 92% | XP_008212974.2 |
| PPU09687 | S229D258H343 | Neutral | Scaffold_30 | 1016596 | 1018949 | PREDICTED: pancreatic triacylglycerol lipase-like isoform X2 [*Nasonia vitripennis*] | 0 | 95% | XP_016844425.1 |
| PPU10689 | S171D195H265 | Neutral | Scaffold_21 | 2421283 | 2426122 | PREDICTED: pancreatic lipase-related protein 2-like [*Nasonia vitripennis*] | 0 | 97% | XP_001600583.1 |
| PPU10799 | S235D260H329 | Neutral | Scaffold_21 | 3702531 | 3707090 | PREDICTED: pancreatic lipase-related protein 2-like [*Nasonia vitripennis*] | 0 | 97% | XP_008217694.1 |
| PPU13285 | S263D296H381 | Neutral | Scaffold_13 | 226949 | 229266 | PREDICTED: pancreatic triacylglycerol lipase-like [*Nasonia vitripennis*] | 0 | 92% | XP_016844594.1 |
| PPU13286 | S111D144H229 | Neutral | Scaffold_13 | 234233 | 236645 | PREDICTED: pancreatic lipase-related protein 2-like [*Nasonia vitripennis*] | 0 | 92% | XP_003424938.1 |
| PPU13290 | S266D295H379 | Neutral | Scaffold_13 | 319585 | 323029 | PREDICTED: pancreatic triacylglycerol lipase [*Nasonia vitripennis*] | 0 | 95% | XP_016843977.1 |
| PPU13747 | S187D211H280 | Neutral | Scaffold_19 | 2214237 | 2215451 | PREDICTED: pancreatic lipase-related protein 2-like [*Nasonia vitripennis*] | 0 | 92% | XP_001605911.2 |
| PPU16276 | S193D220H308 | Neutral | Scaffold_260 | 133224 | 135964 | PREDICTED: pancreatic lipase-related protein 2-like [*Nasonia vitripennis*] | 0 | 96% | XP_008215592.1 |
| PPU16687 | S158D182H244 | Neutral | Scaffold_53 | 789161 | 792351 | PREDICTED: phospholipase A1-like [*Nasonia vitripennis*] | 9.00E-178 | 83% | XP_003426142.1 |
| PPU16688 | S163D187H249 | Neutral | Scaffold_53 | 792719 | 794158 | lipase-like protein precursor [*Nasonia vitripennis*] | 0 | 92% | NP_001164369.1 |
| PPU01585 | S34D62- | Neutral | Scaffold_82 | 268604 | 268955 | PREDICTED: pancreatic lipase-related protein 2-like isoform X2 [*Nasonia vitripennis*] | 5.00E-32 | 79% | XP_016845667.1 |
| PPU01586 | --- | Neutral | Scaffold_82 | 270323 | 270872 | PREDICTED: pancreatic lipase-related protein 2-like isoform X1 [*Nasonia vitripennis*] | 6.00E-17 | 67% | XP_016845666.1 |
| PPU16689 | S191D215H280 | Neutral | Scaffold_53 | 794925 | 796334 | lipase-like protein precursor [*Nasonia vitripennis*] | 0 | 88% | NP_001164368.1 |
| PPU01121 | S194D370H401 | Acid | Scaffold_186 | 343488 | 346195 | PREDICTED: lipase 3-like [Ceratosolen solmsi marchali] | 0 | 82% | XP_011501247.1 |
| PPU01336 | S200D374H405 | Acid | Scaffold_180 | 878988 | 883435 | PREDICTED: lipase 3-like [*Nasonia vitripennis*] | 0 | 77% | XP_016841509.1 |
| PPU01964 | S158D331H362 | Acid | Scaffold_101 | 117745 | 120387 | PREDICTED: lipase 3-like [*Nasonia vitripennis*] | 0 | 90% | XP_001606536.3 |
| PPU01965 | S132D304H335 | Acid | Scaffold_101 | 122004 | 123602 | PREDICTED: lipase member K-like isoform X4 [*Nasonia vitripennis*] | 0 | 83% | XP_001606528.1 |
| PPU01966 | S195D368H399 | Acid | Scaffold_101 | 125427 | 127216 | PREDICTED: lipase 3-like [*Nasonia vitripennis*] | 0 | 82% | XP_016839047.1 |
| PPU10149 | S235D409H440 | Acid | Scaffold_122 | 210120 | 216573 | PREDICTED: lipase 3-like [*Nasonia vitripennis*] | 0 | 92% | XP_008216585.2 |
| PPU10150 | S196D367H397 | Acid | Scaffold_122 | 218387 | 221035 | PREDICTED: lipase 3-like [*Nasonia vitripennis*] | 0 | 89% | XP_008216710.1 |
| PPU10152 | S636D810H841 | Acid | Scaffold_122 | 226530 | 235046 | PREDICTED: lipase 3-like isoform X1 [*Nasonia vitripennis*] | 0.00E+00 | 82% | XP_008216579.1 |
| PPU10153 | S201D379H409 | Acid | Scaffold_122 | 239909 | 245622 | PREDICTED: lipase 3-like [*Nasonia vitripennis*] | 0 | 92% | XP_003424407.1 |
| PPU10154 | S176D350H381 | Acid | Scaffold_122 | 251717 | 257380 | PREDICTED: lipase 3-like [*Nasonia vitripennis*] | 0 | 99% | XP_016838961.1 |
| PPU10157 | S202D376H407 | Acid | Scaffold_122 | 266874 | 268841 | PREDICTED: lipase 3-like [*Nasonia vitripennis*] | 0 | 86% | XP_003424417.1 |
| PPU10158 | S462D637H668 | Acid | Scaffold_122 | 269945 | 278694 | PREDICTED: lipase 3-like isoform X1 [*Nasonia vitripennis*] | 0 | 96% | XP_016840204.1 |
| PPU10155 | S607D781H812 | Acid | Scaffold_122 | 259060 | 263527 | hypothetical protein TSAR_015310 [*Trichomalopsis sarcophagae*] | 0 | 87% | OXU25252.1 |
| PPU10742 | S190D367H398 | Acid | Scaffold_21 | 3082407 | 3084689 | PREDICTED: lipase 3-like [Diachasma alloeum] | 1.00E-126 | 47% | XP_015112844.1 |
| PPU10743 | S314D488H519 | Acid | Scaffold_21 | 3091322 | 3097081 | PREDICTED: gastric triacylglycerol lipase-like isoform X1 [*Nasonia vitripennis*] | 0 | 81% | XP_001602620.2 |
| PPU11414 | S327D335H365 | Acid | Scaffold_26 | 628072 | 631746 | PREDICTED: lipase 3-like [*Nasonia vitripennis*] | 0 | 89% | XP_008211135.2 |
| PPU11430 | S192D362H393 | Acid | Scaffold_26 | 1278560 | 1280365 | PREDICTED: gastric triacylglycerol lipase-like [*Nasonia vitripennis*] | 0 | 87% | XP_016840181.1 |
| PPU11431 | S166D340H370 | Acid | Scaffold_26 | 1280839 | 1282729 | PREDICTED: gastric triacylglycerol lipase-like [*Nasonia vitripennis*] | 0 | 66% |  |
| PPU13507 | S44-- | Acid | Scaffold_13 | 3499463 | 3499764 | PREDICTED: lipase 3-like [*Nasonia vitripennis*] | 4.00E-38 | 87% | XP_008213182.2 |
| PPU13508 | --- | Acid | Scaffold_13 | 3499795 | 3500375 | PREDICTED: lipase 3-like isoform X2 [*Nasonia vitripennis*] | 1.00E-64 | 76% | XP_016840778.1 |
| PPU13511 | S181D354H384 | Acid | Scaffold_13 | 3507498 | 3509575 | PREDICTED: lipase 3-like [*Nasonia vitripennis*] | 0 | 92% | XP_016840785.1 |
| PPU13505 | S167D340H370 | Acid | Scaffold_13 | 3491754 | 3493472 | PREDICTED: lipase 3-like isoform X1 [*Nasonia vitripennis*] | 0 | 88% | XP_016840775.1 |
| PPU13510 | S195D368H398 | Acid | Scaffold_13 | 3503469 | 3506103 | PREDICTED: uncharacterized protein LOC103317300 [*Nasonia vitripennis*] | 0 | 89% | XP_008213053.2 |
| PPU13509 | S132-H287 | Acid | Scaffold_13 | 3500928 | 3502871 | PREDICTED: uncharacterized protein LOC103317300 [*Nasonia vitripennis*] | 6E-177 | 69% |  |
| PPU13932 | S163D337H367 | Acid | Scaffold_18 | 1615834 | 1619062 | PREDICTED: lipase 3-like isoform X1 [*Nasonia vitripennis*] | 0 | 89% | XP_008207853.1 |
| PPU16612 | S241E406D439 | Acid | Scaffold_50 | 903252 | 905522 | lipase A-like precursor [*Nasonia vitripennis*] | 0 | 71% | NP_001154991.1 |
| PPU02316 | S448D668R720 | HSL | Scaffold_361 | 559051 | 562279 | PREDICTED: hormone-sensitive lipase [*Nasonia vitripennis*] | 0 | 92% | XP_016841759.1 |
| PPU02421 | S449E651- | lipase3 | Scaffold_165 | 989475 | 1008762 | PREDICTED: sn1-specific diacylglycerol lipase beta-like isoform X1 [*Nasonia vitripennis*] | 0 | 97% | XP_008209601.1 |
| PPU03672 | S482D675- | lipase3 | Scaffold_1 | 6844639 | 6964488 | PREDICTED: sn1-specific diacylglycerol lipase alpha isoform X6 [*Nasonia vitripennis*] | 0 | 99% | XP_016842279.1 |
| PPU10151 | S78D252H283 | lipase3 | Scaffold_122 | 222168 | 224131 | PREDICTED: lipase 3-like isoform X2 [*Nasonia vitripennis*] | 2E-171 | 75% | XP_016840160.1 |
| PPU11235 | A165M346- | GDSL | Scaffold_25 | 4144782 | 4148568 | PREDICTED: phospholipase B1, membrane-associated [*Nasonia vitripennis*] | 0 | 94% | XP_008210181.2 |
| PPU11239 | S152N331- | GDSL | Scaffold_25 | 4172224 | 4178050 | PREDICTED: phospholipase B1, membrane-associated-like [*Nasonia vitripennis*] | 0 | 96% | XP_008210041.1 |
