# Supplementary material for: Insight into the Functional Diversification of Lipases in the Endoparasitoid Pteromalus puparum (Hymenoptera: Pteromalidae) by Genome-scale Annotation and Expression Analysis
Source: Insects. 2020 Apr 5;11(4):227. doi: 10.3390/insects11040227 (PMC7240578; doi:10.3390/insects11040227)
Supplement: Supplementary file 1 [file insects-11-00227-s001.zip › Supply/Table S4.docx]

| **Table S4. FPKM values of lipases from different libraries of *P. puparum*.** | | | | | | | | | | |
| --- | --- | --- | --- | --- | --- | --- | --- | --- | --- | --- |
| **Tracking-id** | **Embryos** | **larvae** | **Female pupae** | **Male pupae** | **Female adults** | **Male adults** | **Venom gland** | **Carcass** | **Ovary** | **Salivary gland** |
| PPU09230 | 0.0000 | 0.0000 | 0.0000 | 0.0000 | 60.2502 | 0.0000 | 868.8560 | 0.5585 | 4.5289 | 0.0000 |
| PPU11430 | 0.0000 | 1.4014 | 0.0000 | 0.0000 | 14.3239 | 0.3535 | 1041.8000 | 0.9375 | 1.7377 | 0.3816 |
| PPU16612 | 0.0280 | 0.2907 | 0.1117 | 0.0276 | 33.6086 | 0.1213 | 2022.1200 | 2.4425 | 2.6580 | 1.6153 |
| PPU00494 | 0.0000 | 0.0000 | 0.0000 | 0.0000 | 14.4897 | 0.0000 | 1526.2000 | 2.0674 | 0.0000 | 0.0000 |
| PPU04616 | 0.0000 | 0.2072 | 0.0332 | 0.0000 | 4.3302 | 0.0576 | 265.5660 | 0.6116 | 0.0971 | 1.8708 |
| PPU09231 | 0.0000 | 0.0000 | 0.0000 | 0.0501 | 2.3133 | 0.0000 | 211.0410 | 0.5816 | 0.5467 | 0.0000 |
| PPU09658 | 1.2133 | 0.8231 | 1.3079 | 0.4751 | 0.3072 | 1.1853 | 20.2689 | 0.1558 | 1.6661 | 0.8226 |
| PPU01336 | 0.0000 | 175.1420 | 0.8716 | 0.5343 | 0.0000 | 0.0783 | 0.9489 | 0.2767 | 0.2338 | 113.1320 |
| PPU10742 | 0.0928 | 13.3692 | 0.0000 | 0.2441 | 0.0536 | 0.0536 | 0.3483 | 0.1422 | 0.1503 | 0.5173 |
| PPU10799 | 1.5381 | 1.3610 | 2.1611 | 3.1365 | 13.1676 | 6.7264 | 10.1819 | 5.3477 | 1.2109 | 1.9926 |
| PPU09657 | 28.1952 | 21.0294 | 18.6189 | 7.8266 | 49.1589 | 25.0255 | 37.3270 | 25.6574 | 24.8762 | 15.2173 |
| PPU13747 | 0.1169 | 0.0000 | 0.4308 | 0.2321 | 0.9771 | 0.5411 | 0.9690 | 0.9898 | 0.0382 | 0.1190 |
| PPU05374 | 0.0810 | 0.2533 | 0.1631 | 0.4831 | 0.7399 | 0.4924 | 0.2749 | 0.2809 | 0.3183 | 0.3096 |
| PPU02421 | 0.6808 | 2.8557 | 47.0836 | 20.1512 | 13.2276 | 7.2240 | 4.2831 | 4.8286 | 4.7759 | 0.6663 |
| PPU08363 | 0.2641 | 4.5656 | 3.8656 | 4.4047 | 5.4992 | 4.3808 | 2.9032 | 3.9518 | 5.1772 | 2.5633 |
| PPU13932 | 5.9161 | 1.9691 | 0.3385 | 0.7021 | 21.6926 | 34.6618 | 20.2088 | 34.4128 | 4.1858 | 3.1410 |
| PPU01966 | 4.5890 | 23.6796 | 32.0236 | 39.5315 | 69.6886 | 54.1014 | 17.9136 | 31.9960 | 35.0042 | 13.4403 |
| PPU06393 | 0.0000 | 0.6762 | 0.4791 | 1.0642 | 8.5703 | 1.6859 | 4.0167 | 9.1796 | 0.8236 | 0.0000 |
| PPU02316 | 3.8304 | 7.2073 | 4.4973 | 8.8841 | 17.9124 | 7.9866 | 2.0218 | 7.5691 | 11.2175 | 15.2128 |
| PPU10158 | 0.0531 | 8.5167 | 3.1682 | 4.6852 | 18.9775 | 8.4968 | 1.3866 | 5.6138 | 0.4236 | 1.5222 |
| PPU13285 | 0.0000 | 11.6812 | 0.0263 | 0.0260 | 0.8888 | 0.3434 | 0.1188 | 0.4848 | 0.2555 | 6.6525 |
| PPU13286 | 0.0403 | 0.0841 | 0.0000 | 0.1604 | 86.8444 | 42.5409 | 16.3449 | 69.4941 | 8.6005 | 0.0000 |
| PPU09687 | 0.1164 | 0.0000 | 0.1451 | 0.0860 | 0.0509 | 0.0000 | 0.0655 | 0.3341 | 0.0564 | 0.0000 |
| PPU10149 | 0.1090 | 1.1882 | 0.2173 | 3.6207 | 8.9878 | 16.6496 | 0.8581 | 5.4408 | 3.6401 | 1.7370 |
| PPU13511 | 0.0000 | 0.1326 | 0.0638 | 0.0000 | 2.9725 | 0.6084 | 0.2875 | 1.9075 | 10.5170 | 0.0970 |
| PPU10151 | 0.0000 | 0.2832 | 0.0000 | 0.0000 | 1.6186 | 0.5109 | 0.2051 | 1.3626 | 0.0447 | 0.0000 |
| PPU09686 | 0.0000 | 0.2548 | 0.0106 | 0.0174 | 0.0000 | 0.1605 | 2.0366 | 13.5565 | 0.0171 | 0.0269 |
| PPU10743 | 0.0000 | 0.7218 | 0.1153 | 0.1138 | 8.8432 | 12.9417 | 0.5209 | 3.6128 | 0.9391 | 33.0607 |
| PPU06272 | 4.4495 | 0.6664 | 0.6010 | 1.3330 | 25.5399 | 27.4259 | 1.2856 | 9.0644 | 4.9078 | 0.5023 |
| PPU10152 | 0.0000 | 0.1136 | 0.0136 | 0.0000 | 9.0263 | 4.8412 | 0.3992 | 4.0366 | 0.0131 | 0.0000 |
| PPU07195 | 0.0000 | 0.0000 | 0.0616 | 0.1217 | 6.2585 | 0.0267 | 0.1389 | 1.6309 | 11.0581 | 0.0000 |
| PPU01965 | 1.9756 | 14.0895 | 6.8125 | 5.1748 | 11.0529 | 25.9488 | 0.4212 | 4.9893 | 2.9919 | 3.4706 |
| PPU11431 | 0.0335 | 37.0046 | 0.0000 | 0.2650 | 0.8611 | 3.1968 | 0.3023 | 3.6260 | 0.1306 | 5.7656 |
| PPU10689 | 0.1241 | 24.2514 | 21.4310 | 19.0209 | 76.1445 | 52.0563 | 1.6868 | 20.5839 | 9.2394 | 3.7362 |
| PPU13290 | 53.2976 | 0.1681 | 10.8100 | 10.3838 | 38.2233 | 4.0697 | 2.8527 | 39.8821 | 42.1521 | 0.2867 |
| PPU07948 | 0.1228 | 2.7312 | 0.0825 | 0.0407 | 2.8502 | 4.7644 | 0.4633 | 6.5322 | 9.2948 | 24.1077 |
| PPU03672 | 1.6612 | 1.4367 | 3.3747 | 3.5669 | 5.2600 | 2.4300 | 0.1785 | 2.6935 | 2.6395 | 1.3478 |
| PPU01121 | 172.7690 | 15.8404 | 18.6917 | 16.7343 | 53.9861 | 26.4068 | 3.7999 | 63.7464 | 50.8069 | 11.2391 |
| PPU01964 | 0.0335 | 3.1708 | 1.3753 | 1.2919 | 27.7857 | 24.1790 | 1.0580 | 22.9131 | 6.8572 | 0.1531 |
| PPU13510 | 0.9987 | 11.7584 | 0.0907 | 0.0298 | 4.0521 | 2.9119 | 0.0681 | 3.1298 | 2.0863 | 0.8739 |
| PPU11414 | 0.0678 | 0.2821 | 0.3396 | 0.4695 | 24.0097 | 34.1492 | 0.1530 | 20.6955 | 0.1653 | 60.2503 |
| PPU16688 | 0.0000 | 9.0442 | 2.1756 | 8.2492 | 27.6913 | 10.5312 | 0.0977 | 34.9566 | 0.0000 | 43.6674 |
| PPU03462 | 0.2364 | 0.5564 | 1.0441 | 1.2206 | 0.7786 | 1.3760 | 0.0000 | 0.2948 | 0.6609 | 0.0650 |
| PPU03466 | 0.0468 | 0.8977 | 7.6949 | 11.1356 | 5.1687 | 11.7327 | 0.0000 | 6.6436 | 0.0902 | 0.1771 |
| PPU05625 | 0.0000 | 2.2812 | 0.6951 | 0.4576 | 0.7728 | 0.4336 | 0.0000 | 0.7098 | 1.5439 | 0.2934 |
| PPU06394 | 0.0336 | 0.0000 | 0.0000 | 0.0000 | 26.7050 | 35.1532 | 0.0000 | 48.0550 | 0.0000 | 0.0000 |
| PPU10153 | 0.6458 | 6.3537 | 1.0355 | 4.4223 | 0.7890 | 0.2919 | 0.0000 | 0.7733 | 0.1903 | 1.1506 |
| PPU10154 | 0.1287 | 0.0334 | 0.3860 | 1.9696 | 0.2772 | 0.5857 | 0.0000 | 0.2960 | 0.0000 | 0.7342 |
| PPU10155 | 0.0145 | 0.1046 | 0.0998 | 0.1267 | 0.0542 | 0.0250 | 0.0000 | 0.0329 | 0.0000 | 360.4350 |
| PPU10157 | 0.2408 | 0.2502 | 0.1803 | 0.2671 | 0.8905 | 0.9132 | 0.0000 | 4.0812 | 0.1753 | 273.7940 |
| PPU11235 | 0.1892 | 0.0983 | 0.5043 | 0.5603 | 2.3443 | 1.9686 | 0.0000 | 2.2483 | 0.1840 | 0.0000 |
| PPU11239 | 3.3648 | 0.2941 | 0.2829 | 0.3104 | 1.9579 | 0.2999 | 0.0000 | 0.4339 | 1.6816 | 0.0957 |
| PPU13505 | 0.0655 | 0.0681 | 0.0000 | 0.0000 | 0.7315 | 4.3140 | 0.0000 | 0.6026 | 0.6373 | 0.0996 |
| PPU13509 | 0.0000 | 0.0000 | 0.0000 | 0.0000 | 0.4276 | 0.1888 | 0.0000 | 0.0000 | 0.2571 | 0.0666 |
| PPU16276 | 0.4874 | 0.3790 | 0.4118 | 1.0526 | 0.9581 | 0.4220 | 0.0000 | 0.9488 | 1.6452 | 1.5132 |
| PPU16687 | 0.0251 | 0.4681 | 0.0499 | 0.0000 | 0.8039 | 2.6926 | 0.0000 | 2.8151 | 0.0484 | 28.0762 |
| PPU16689 | 0.2297 | 2.2734 | 0.2694 | 1.8619 | 6.6146 | 1.8948 | 0.0000 | 30.0612 | 0.3377 | 1927.6300 |
| PPU01585 | 0.0000 | 0.0000 | 0.4211 | 0.8255 | 1.4989 | 0.0000 | 107.4100 | 0.0000 | 0.0000 | 0.0000 |
| PPU01586 | 2.8736 | 0.0000 | 0.0000 | 1.9553 | 2.5260 | 0.0000 | 50.5857 | 0.0000 | 0.7064 | 0.0000 |
| PPU04615 | 0.0000 | 0.0000 | 0.0000 | 0.0000 | 4.9421 | 0.0000 | 47.8084 | 0.0000 | 0.2595 | 0.0000 |
| PPU06103 | 0.0000 | 0.0000 | 0.0000 | 0.8393 | 0.0000 | 0.0733 | 0.0955 | 0.0000 | 0.0000 | 0.0000 |
| PPU10150 | 0.0000 | 62.3266 | 0.0000 | 0.3060 | 0.3762 | 0.1344 | 1.1178 | 0.0000 | 0.0000 | 24.8997 |
| PPU13507 | 0.0000 | 0.0000 | 0.7412 | 0.0000 | 0.2952 | 0.6467 | 0.0000 | 0.0000 | 1.7016 | 0.0000 |
| PPU13508 | 0.0000 | 0.0000 | 0.0000 | 0.0000 | 0.0000 | 0.1387 | 0.0000 | 0.0000 | 1.9200 | 0.0000 |
