# Supplementary figures and images for: Insight into the Functional Diversification of Lipases in the Endoparasitoid Pteromalus puparum (Hymenoptera: Pteromalidae) by Genome-scale Annotation and Expression Analysis
Source: Insects. 2020 Apr 5;11(4):227. doi: 10.3390/insects11040227 (PMC7240578; doi:10.3390/insects11040227)

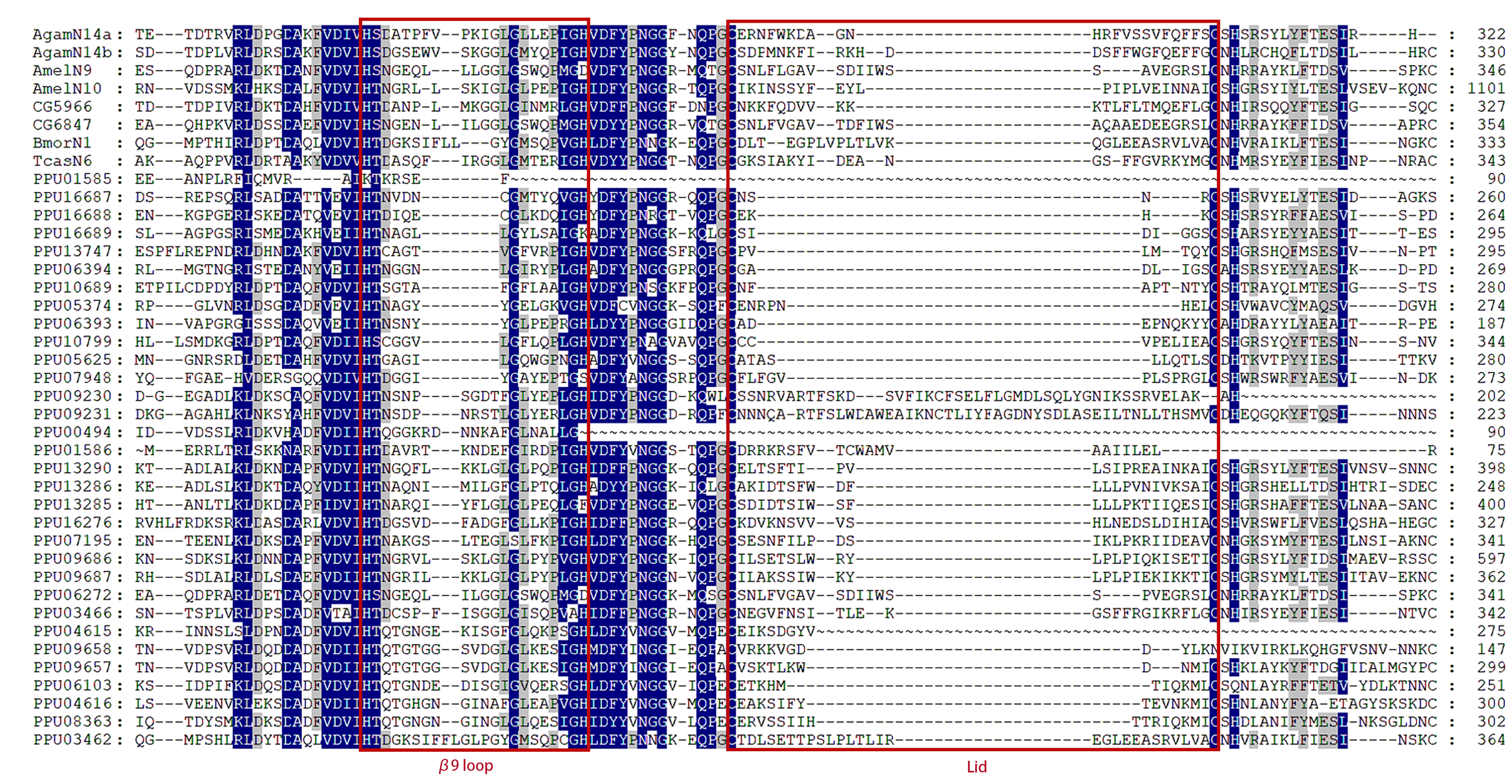

Supplement: Supplementary file 1 [file insects-11-00227-s001.zip › Supply/Figure S1.tif]
